# The anti-inflammatory properties of *Satureja khuzistanica* Jamzad essential oil attenuate the effects of traumatic brain injuries in rats

**DOI:** 10.1038/srep31866

**Published:** 2016-08-18

**Authors:** Elham Abbasloo, Fatemeh Dehghan, Mohammad Khaksari, Hamid Najafipour, Reza Vahidi, Shahriar Dabiri, Gholamreza Sepehri, Golamreza Asadikaram

**Affiliations:** 1 Physiology Research Center, Institute of Neuropharmacology, Kerman University of Medical Sciences, Kerman, Iran; 2 Department of physiology, Fasa University of Medical Sciences, Fasa, Iran; 3 Endocrinology and Metabolism Research Center, Institute of Basic and Clinical Physiology Sciences, Kerman University of Medical Sciences, Kerman, Iran; 4 Cardiovascular research Center, Institute of Basic and Clinical Physiology Sciences, Kerman University of Medical Sciences, Kerman, Iran; 5 Pathology and Stem Cell Research center, Kerman University of Medical Sciences, Kerman, Iran; 6 Neuroscience Research Center, Institute of Neuropharmacology, Kerman University of Medical Sciences, Kerman, Iran

## Abstract

Traumatic brain injury (TBI) is a major health concern affecting the general public as well as military personnel. However, there is no FDA-approved therapy for the treatment of TBIs. In this work, we investigated the neurotherapeutic effects of the well-known natural Iranian medicine *Satureja Khuzistanica* Jamzad (SKJ) essential oil (SKEO) on the outcomes of diffused experimental TBI, with particular attention paid to its anti-inflammatory and anti-apoptotic effects. Male Wistar rats were treated with doses of 50, 100 and 200 (mg/kg, i.p) SKEO after induction of diffused TBIs. The results showed that injecting SKEO (200 mg/kg) 30 minutes after TBI significantly reduced brain oedema and damage to the blood-brain barrier (BBB) and limited the post-TBI increase in intracranial pressure. The veterinary coma scale (VCS) scores significantly improved in the treatment group. Also, inflammatory marker assays showed reduced levels of TNF-α, IL-1β, and IL-6 and increased IL-10 in the treated groups. Moreover, the immunohistochemical results indicated that SKEO not only reduced neuronal death and BBB permeability but also affected astrocytic activation. Overall, our data indicate potential clinical neurological applications for SKEO.

Traumatic brain injury (TBI) imposes a substantial medical burden worldwide. There are an estimated 1.7 million annual TBI-related injuries in the US, including 52,000 deaths^1^. A neurotherapeutic intervention to treat TBI-related injuries is greatly needed^2^. The damage caused by TBIs results from injuries to axons, blood vessels, and glial cells^3^. TBIs increase the permeability of the blood-brain barrier (BBB) and consequently increase brain oedema and intracranial pressure (ICP)^4^.

Thus far, the treatment methods used for TBIs have focused on reducing intracranial pressure and preventing oxidative stress after injury. An effective pharmacological treatment for TBI has yet to be found^2^,^4^.

Minutes after a traumatic impact, a severe inflammatory response is elicited in the injured brain. The complex post-traumatic process involves a cellular component comprising the activation of resident glial cells, microglia, and astrocytes^5^ and the infiltration of blood leukocytes^6^. A second component is related to the secretion of immune mediators: the archetypal pro-inflammatory cytokines (Interleukin-1, Tumour Necrosis Factor-α, and Interleukin-6), the anti-inflammatory cytokines (Interleukin-4, Interleukin-10, and TGF-β), and the chemotactic cytokines or chemokines, which specifically drive the accumulation of parenchymal and peripheral immune cells to the injured brain region^7^. These mechanisms have been demonstrated in both animal and human brains^8^,^9^.

The use of medicinal herbs is common worldwide. Promoting the rational use of herbal medicine through accurate technical guidelines and international standards has become the policy of the World Health Assembly (WHA)^10^. Medicinal herbs often have few side effects and are multi-functional^11^. The genus *Satureja* is mainly found in the Mediterranean region and belongs to the Lamiaceae family and the Nepetoidae subfamily; 14 species grow in Iran^12^. *Satureja khuzistanica* Jamzad (SKJ) (also called *Marzeh khuzestani* in Persian) is one of the species that grows in the southern and south western parts of Iran. This plant has been used in traditional Iranian medicine for its analgesic and antiseptic effects^13^. The medical properties of this plant are well-known among the nomadic inhabitants of the south western part of Iran^14^. Furthermore, *Satureja khuzistanica* Jamzad essential oil (SKEO) is used in the pharmaceutical and food industries as a dental anaesthetic and in oral antiseptic drops^12^. There is evidence to support the anti-apoptotic, anti-allergic, neuroprotective^15^, and anti-inflammatory effects of its extract. Anti-oxidant^16^ and anti-inflammatory effects of SKEO have also been reported^17^. The plant contains more than 4.5% essential oil^12^, and carvacrol is the most abundant compound in SKEO (94.16%) (Table 1)^18^,^19^. Due to the low molecular mass and lipophilic properties of this compound, it can easily cross the BBB^20^.

In this study, we assessed the possible protective effects of SKEO in an animal model of TBI. We studied the effect of SKEO on damage to the BBB, brain oedema, and increased ICP, along with its effects on inflammatory processes and neuronal activation, as assessed by immunohistochemistry (IHC).

## Materials and Methods

### Experimental procedures

#### Animals

The study was performed in accordance with NIH guidelines. The experimental protocol was approved by the ethical committee of the Kerman University of Medical Sciences, Kerman, Iran (Ethic code No 92/330KA). Animals (male Wistar rats, weighing 200–250 g) were housed in an air-conditioned room at 22–25 °C with a 12 hr light/dark cycle. All animals had free access to food and water.

#### Preparing the essential oil

SKJ was collected from a cultivated source (Khorraman Farm, Khorramabad, Iran) during the flowering stage of the plant and identified by the Department of Botany of the Research Institute of Forests and Rangelands (TARI), Tehran, Iran. A voucher specimen (No. 58416) was deposited at the Herbarium of TARI. The collected materials were air-dried, crushed, boiled for 5 hr in distilled water using a Clevenger machine, and distilled. The water and the evaporated essence mixture were separated due to the difference in mass between the water and the essence. The acquired yellow oil (essence) was separated, and the suspended water particles were absorbed using a sodium sulphate solution. Gas chromatography-mass spectroscopy (GC-Mass) analysis revealed that carvacrol (94.16%), p-Cymene (0.96%) and γ-Terpenene (0.51%) composed the majority of the essence (Table 1)^18^. The essential oil was diluted in 1% Tween 20 and administered 30 minutes after TBI.

Doses of 50, 100, and 200 mg/kg were selected in accordance with the study by Abdollahi *et al.*^13^, who determined that doses of SKEO of up to 2,000 mg/kg are non-toxic. However, in a pilot experiment, we observed sleepiness in the experimental animals that had received doses of more than 200 mg/kg SKEO. Amanlou *et al.* also reported that the intraperitoneal administration of doses of more than 200 mg/kg of SKJ was associated with drowsiness and reduced physical activity in rats^16^. Therefore, we avoided using higher doses due to the potential for interference with variables such as neurological outcomes (e.g., VCS) that are consciousness-dependent.

#### Experimental protocols

**Figure 1:**
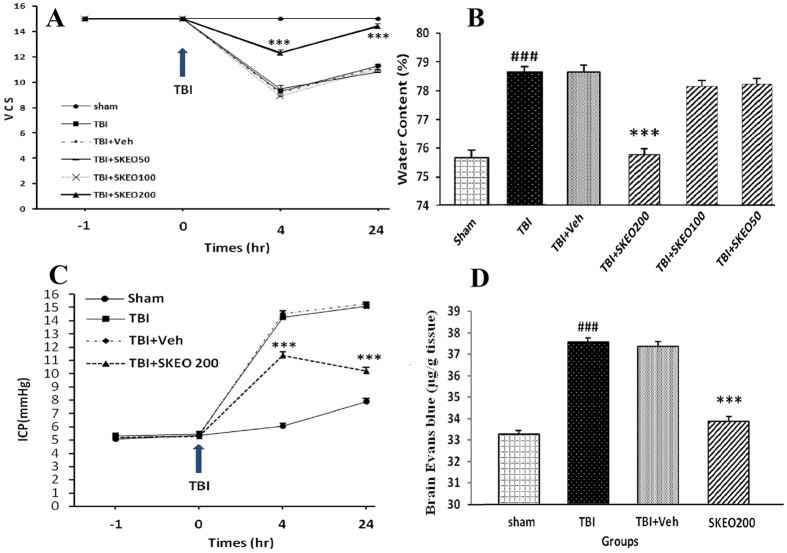
The effect of SKEO on TBI parameters in the rat brain. (**A**) Changes in veterinary coma scale (VCS) scores over time in different groups. ***p < 0.001 at 4 and 24 hours (hr) between TBI+SKEO200 and the other groups. TBI decreased the VCS scores significantly at 4 and 24 hr in all the groups. However, SKEO200 caused greater improvement in the VCS scores compared to the other treatments. (**B**) Brain water content after TBI in the different groups. ^###^p < 0.001 vs. sham, ***p < 0.001 vs. TBI+Veh. (**C**) Intracranial pressure (ICP) in the different groups. ***p < 0.001 at 4 and 24 hr after TBI for the TBI+SKEO200 group compared to the TBI+Veh group. (**D**) Evans blue dye content (μg/g tissue); ^###^p < 0.001 in the TBI group compared to the sham group. ***p < 0.001 in the TBI+ SKEO 200 group compared to the TBI+Veh group. The data in all figures are means ± SEM (n = 7 in each group).

The animals were randomly divided into six main groups that were each subdivided into five subgroups (n = 7), and measurements of neurological score and brain water content (subgroup 1), Evans blue dye content (subgroup 2), intracranial pressure (subgroup 3), cytokine levels including TNF-α, IL-1β, IL-6, and IL-10 (subgroup 4), and pathological scores (H&E and immunohistochemical staining; subgroup 5) were taken. Based on the data collected from veterinary coma scale (VCS) and water content (WC) measurements (Fig. 1A,B), doses of 50 and 100 mg/kg of SKEO were found to be ineffective, whereas a dose of 200 mg/kg was found to significantly reduce VCS and WC.

Therefore, the remaining experiments were conducted using a dose of 200 mg/kg SKEO. This resulted in a reduction in the number of animals used and in other research expenses.

The six main groups were as follows:

1. Sham: these rats underwent preparatory procedures for brain trauma but were not exposed to brain trauma.
2. TBI: these rats were exposed to brain trauma and received no treatment.
3. TBI+Veh: these rats received an intraperitoneal (i.p) injection of vehicle (tween20, 1%) 30 minutes after TBI induction^21^.
4. TBI+SKEO50: these rats received SKEO (50 mg/kg, i.p) 30 minutes after TBI induction. (Only VCS and WC measurements were performed in this group).
5. TBI+SKEO100: these rats received SKEO (100 mg/kg, i.p) 30 minutes after TBI induction. (Only VCS and WC measurements were performed in this group).
6. TBI+SKEO200: these rats received SKEO (200 mg/kg, i.p) 30 minutes after TBI induction.

#### Induction of diffuse traumatic brain injury (TBI)

All animals were intubated before TBIs were administered. The TBI method used was moderate and diffuse, as induced using the Marmarou method^22^,^23^ using a TBI device made by the Department of Physiology, Kerman University of Medical Sciences.

The protocol was as follows: a 300 g weight was dropped from a 2 m height onto the head of the anaesthetized rat (receiving a gas mixture of isoflurane/N2O/O2; 2%/66%/33%, respectively) while a metal disc (stainless steel, 10 mm in diameter, 3 mm thick) was attached to the animal’s skull. After induction of the trauma, the rats were immediately connected to a respiratory pump (TSA animal respiratory compact, Germany). After spontaneous breathing recovered, the intra-tracheal tube was removed and the rats were placed in individual cages.

#### Determination of the brain’s water content (WC)

Brain oedema was assessed by measuring the water content of the brain. Anaesthetized animals were sacrificed 24 hr after the TBI and the brain was removed, placed in pre-weighed glass vials, and weighed (wet weight). The vials were placed in an incubator (Memmert, Germany) at 60 °C for 72 hr, and then weighed again (dry weight). The percentage of water in each sample was then calculated using the formula^23^:


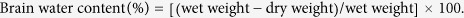


#### Determination of blood–brain barrier (BBB) permeability

According to O’Connor’s protocol^23^, 4 hr after the TBI a dose of 20 ml/kg Evans blue (EB) was injected intravenously through the tail vein. One hour later, animals were anaesthetized and intravascular EB dye was washed out by saline infusion. Animals were then decapitated and their brains were removed and homogenized in phosphate buffered saline. Protein was precipitated by the addition of trichloroacetic acid, cooled and centrifuged (at 2,000 cycles/min for 10 min). The resulting supernatant was used to measure the EB absorbance at 610 nm using a spectrophotometer (UV/VIS, Spectrometer, UK). The following formula was used to calculate the EB dye content:


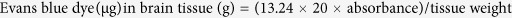


Larger amounts of dye in the brain tissue represent more severe BBB disruption^23^.

#### Evaluation of intracranial pressure

Intracranial pressure was determined by an ICP monitoring system (Mobin Kahroba Kimia Co. Kerman, Iran). Before measuring the ICP, animals were anaesthetized (with a gas mixture of isoflurane/N2O/O2) and placed in stereotactic instruments with their heads in the mid-sagittal plane and the anterior–posterior point located midway between the occipital crest and the lambda suture. After identifying the cisterna magna^24^, a 20-gauge needle connected to a saline-filled PE50 tube was inserted into the cisterna magna to transfer pressure to the pressure transducer of the ICP monitoring system. The pressure was recorded before induction of the trauma and at 4 and 24 hr after induction of TBI^25^,^26^.

#### Evaluation of neurological outcomes

Neurological outcomes were assessed based on the veterinary coma scale (VCS), which is scored from 3–15, and is the sum of the motor response score (1–8), the visual response score (1–4) and the respiratory response score (1–3). A higher score indicates better neurological outcomes. The VCS was determined before induction of the trauma and at 4 and 24 hr after induction of the TBI^27^.

#### Measurements of brain cytokines

Twenty-four hours after induction of the TBI, rats were anaesthetized with sodium thiopental (50 mg/kg, i.p) and their brains were removed and immediately frozen in liquid nitrogen. The brains were weighed and homogenized in T-PERTM tissue protein extraction reagent with 0.5% Triton-x100, 150 mMNaCl, 50 mMTris, and a protease inhibitor cocktail (1 ml in 10 ml of reagent). Following homogenization, the samples were shaken for 90 min and then centrifuged (Rotina, Germany) at 4,000 rpm at 4 °C for 15 min. The supernatant was collected for the analysis^28^ of IL-1β, IL-6, TNF-α and IL-10 using ELISA kits (eBioscience, USA) according to the manufacturer’s guidelines. The concentrations of the cytokines were quantified as pg/100 mg tissue.

#### Immunohistochemistry (IHC)

To determine astrocytic and neuronal activation, glial fibrillary acidic protein (GFAP) and neuron-specific enolase (NSE) levels were assessed^29^. Similarly, CD68 protein (expressed in phagocytosing macrophages and/or reactive microglia)^30^, along with CD3 (lymphocyte markers) levels were assessed^31^ using a Leica DM500-Germany microscope attached to a ICC50 HD digital camera. Positive IHC-stained cells were counted in five different high power fields (HPF; 400X).

### Preparation of slides

Twenty-four hours after injury, animals were deeply anaesthetized with thiopental (50 mg/kg, i.p) and their brains were quickly removed, fixed overnight in 10% formalin, and routinely dehydrated. A cortical coronal slice containing the trauma site was embedded in paraffin, serially sectioned (4 μm), and then dewaxed. After dewaxing, slides were boiled (in a 600 W microwave oven) for 10 min at 120 °C. The slides were incubated at room temperature for 20 min and then washed in phosphate-buffered saline (PBS), exposed to hydrogen peroxide 0.03%, and washed in PBS.

Primary antibodies for NSE, CD3, and CD68 (purchased from DAKO, ready-to-use), and GFAP (1:100, Abcam) were added to the slides at room temperature for 60 min. Secondary antibody (HRP Rabbit/Mouse k5007-DAKO) was then added, after which the slides were washed in PBS. DAB chromogen (Diaminobenzidine Tetrahydrochloride, Sigma USA) was added, and the slides were counter-stained with haematoxylin. The slides were then placed in ascending concentrations of alcohol and then xylene was added. Finally, the sections were mounted with entella. To histologically evaluate the lesions caused by brain trauma, additional sections were stained with haematoxylin and eosin (H&E).

### Statistics

Data normality was assessed using Shapiro Wilk’s W test. A mixed-design analysis of variance was used to evaluate any interactions between the time points of ICP and VCS measurements among groups. When sphericity was violated, a Green-Geisser correction was applied and the data were analysed using one-way ANOVA. Data on water content, EB, and cytokines were analysed using one-way ANOVA followed by an HSD test for post hoc analysis. The differences among groups in the IHC study were analysed using Tukey-Scheffé post hoc tests. P values of less than 0.05 were considered significant. The results are expressed as the mean ± SEM.

## Results

Based on the dose-response study (50–200 mg/kg), we found that doses of 50 and 100 mg/kg SKEO were ineffective, while a dose of 200 mg/kg improved VCS at 4 and 24 hr after TBI (Fig. 1A) and significantly reduced brain water content (Fig. 1B).

### Neurological Outcome

Figure 1A illustrates changes in VCS in different groups at different post-TBI time points. The VCS scores were not significantly different among all groups before trauma was induced. The VCS scores decreased in all trauma groups 4 and 24 hr after the trauma, but there was a significant difference in the TBI+SKEO200 group in comparison with the TBI+Veh group (p < 0.001). Although the TBI and TBI+Veh groups exhibited improvements in their neurological scales 24 hr after TBI, the TBI+SKEO200 group showed a significant improvement compared to the TBI+Veh group (p < 0.001). There was no significant difference in VCS between the TBI+SKEO50 and TBI+SKEO100 groups compared to the TBI and TBI+Veh groups at any time after TBI.

### Brain oedema

Changes in brain water content 24 hr post-TBI are shown in Fig. 1B. The brain water content increased in the TBI group in comparison with the sham group (p < 0.001). The brain water content in the TBI+SKEO200 group significantly decreased in comparison with the TBI+Veh group (p < 0.001), whereas no significant differences were observed in the water content between the TBI and TBI+Veh groups. SKEO50 and SKEO100 treatments did not have significant effects on WC.

### ICP

Changes in the ICPs of all studied groups at different times after TBI are shown in Fig. 1C. No significant differences in the ICPs were observed among the groups before TBI. The induction of trauma resulted in an increase in ICP in all trauma groups 4 and 24 hr after trauma, and the TBI+SKEO200 group showed a significant reduction in ICP at 4 and 24 hr after TBI in comparison with the TBI+Veh group (p < 0.001). There was no significant difference in ICP between the TBI and TBI+Veh groups.

### BBB permeability

Figure 1D shows the EB dye content in all groups 24 hr after TBI. The EB dye content in the TBI group was significantly higher than in the sham group (p < 0.001). The TBI+SKEO200 group showed a significant decrease in EB dye content in comparison with the TBI+Veh group (p < 0.001), while the EB content was not significantly different between the TBI and TBI+Veh groups.

### Cytokine levels

**Figure 2:**
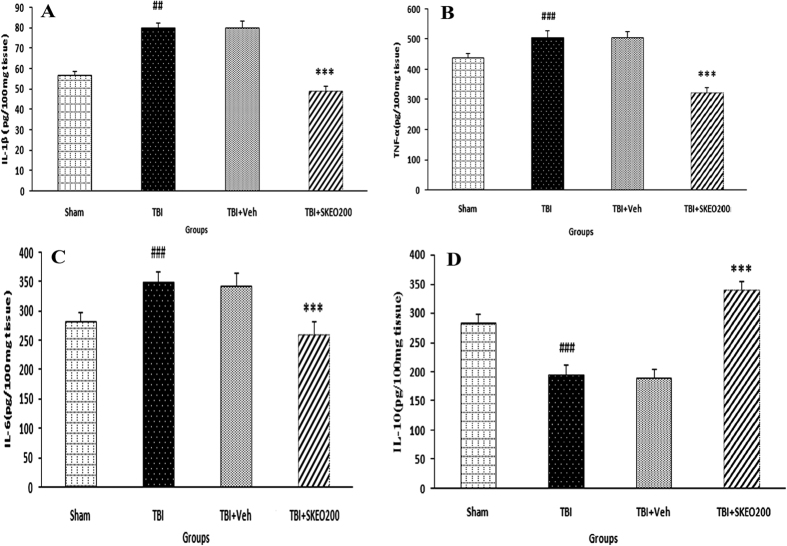
Brain cytokine levels: (**A**) IL-1β, (**B**) TNF-α, (**C**) IL-6, and (**D**) IL-10 levels 24 hr after TBI. ***p < 0.001 compared to the TBI+Veh group. ^###^p < 0.001, ^##^p < 0.01 compared to the sham group (n = 7 in each group).

Figure 2A shows a decrease in IL-1β levels in the TBI+SKEO200 group, which are significantly different from the TBI+Veh group (P < 0.001). A significant difference was also observed between the TBI and sham groups (P < 0.01), but no significant differences were observed between the TBI and TBI+Veh groups. TNF-α levels also significantly decreased in the SKEO200 group (p < 0.001), but no significant difference was found between the TBI and TBI+Veh groups (Fig. 2B). A significant difference in TNF-α levels was found between the TBI and sham groups (P < 0.001). Figure 2C shows a significant difference in IL-6 levels between the TBI and sham groups (P < 0.001). SKEO significantly decreased the level of IL-6 in the TBI+SKEO200 group compared to the TBI+Veh group (P < 0.001), whereas the difference between the TBI and TBI+Veh groups was not significant.

IL-10 levels are shown in Fig. 2D. A significant difference was found between the TBI and sham groups (P < 0.001). The IL-10 level increased after the administration of SKEO200 and there was a significant difference in the TBI+SKEO200 group compared to the TBI+Veh group (p < 0.001).

### Histological and Immunohistochemistry findings

**Figure 3:**
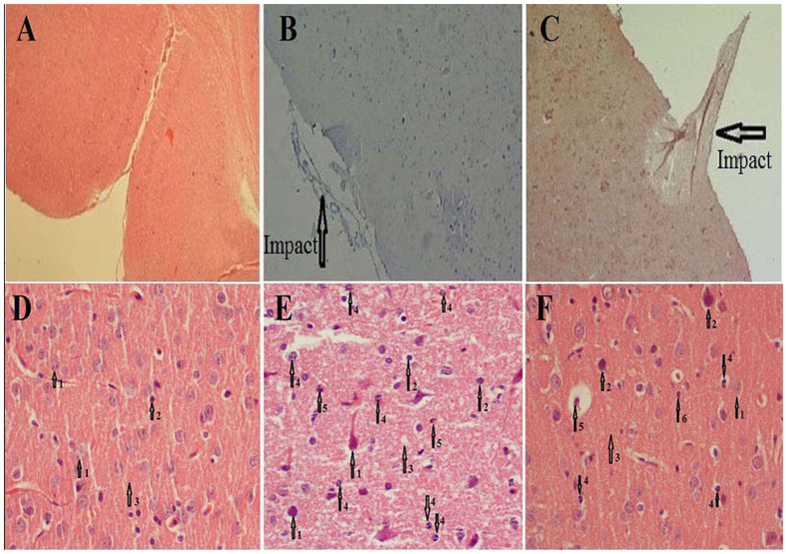
H&E staining micrographs from the sham, TBI, and TBI+SKEO200 groups. Upper row: 40X Lower row: 400X. Black arrows indicate the impact of TBI. (**D**) Brain stoma in a normal rat; numbered arrows indicated the neurons (1), astrocyte (2) and parenchyma (3). (**E**) TBI group; numbered arrows: degeneration of neurons (1), astrocytes (2), reticular parenchyma with oedema (3), neutrophils (4) and eosinophils (5). (**F**) TBI+SKEO200; more neuronal structures and brain parenchyma remained normal; numbered arrows: neuron (1), degeneration of neurons (2), parenchyma (3), neutrophils (4), eosinophils (5) and astrocyte (6).

**Figure 4:**
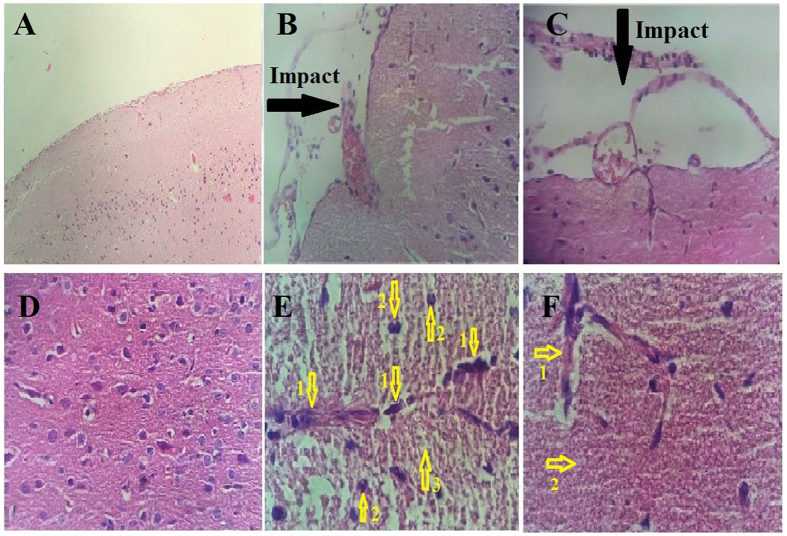
H&E staining micrographs from the sham, TBI and TBI+SKEO200 groups at 24 hr after TBI. Upper row: (**A**: 40X, **B,C**: 100X) Lower row: 1000X. Black arrows show the impact of TBI. (**D**) The normal brain without any pathological destruction. (**E**) Brain stoma in the TBI group; numbered arrow indicate the discontinuity of vessels (2) accumulation of water (oedema) in the brain parenchyma (3), and neutrophils infiltration (3). (**F**) Improved continuity of vessels and parenchyma in the TBI+SKEO200 group; numbered arrows indicate the vessel (1) and parenchyma (2).

**Figure 5:**
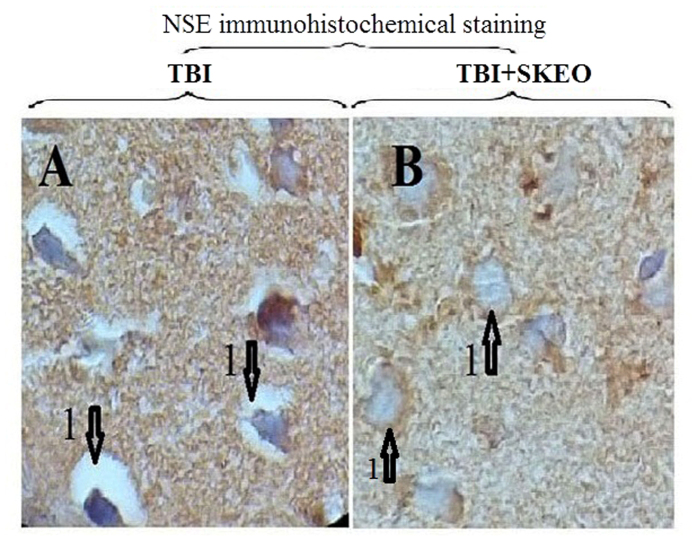
NSE immunohistochemical staining showing vacuolization and synergetic oedema in rat brain cells 24 hours after TBI, (1000X). (**A**) Neuronal vacuolization in the post-TBI brain in TBI group; numbered arrows indicate the synergetic oedema (1). (**B**) TBI+SKEO200; numbered arrows: normal neurons (1).

Brain tissue oedema was observed in the TBI group (Fig. 3E), and the brain vessels were engorged after the TBI. However, the brain vessels in the TBI+SKEO200 group were tightened. Our data also show an obvious reduction of oedema in the brain parenchyma in the TBI group after treatment with SKEO200 (Fig. 4). Neuronal vacuolization was also observed in the TBI group but was not present in the TBI+SKEO200 group (Fig. 5).

**Figure 6:**
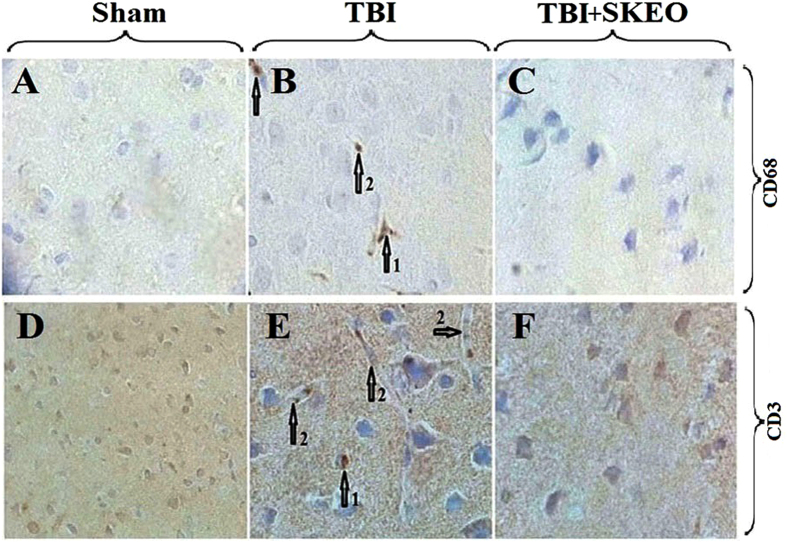
Upper row: IHC staining for CD68-macrophages in the sham, TBI and TBI+SKEO200 groups at 24 hr after TBI (400X); numbered arrows indicate the macrophage (1), microglia (2) and non-activated macrophage in the vessel (3). Lower row: IHC staining for CD3-lymphocytes in the sham, TBI and TBI+SKEO200 groups at 24 hr after TBI (400X); arrows: lymphocytes (1) are only present in the vessels (2) of the TBI group.

The percentage of neutrophils in TBI+SKEO200 group (4.64 ± 0.61%) was significantly lower than it in the brain stoma in the TBI group (14 ± 3.07%, Fig. 3) (p < 0.001). The percentage of macrophages in the TBI+SKEO200 group (0.98 ± 0.25%) was significantly lower than it in the brain stoma in the TBI group (15.25 ± 2.6%, Fig. 6) (p < 0.001). No CD3+ T-cell was found in the brain stoma of both the sham and TBI+SKEO200 groups (Fig. 6).

**Figure 7:**
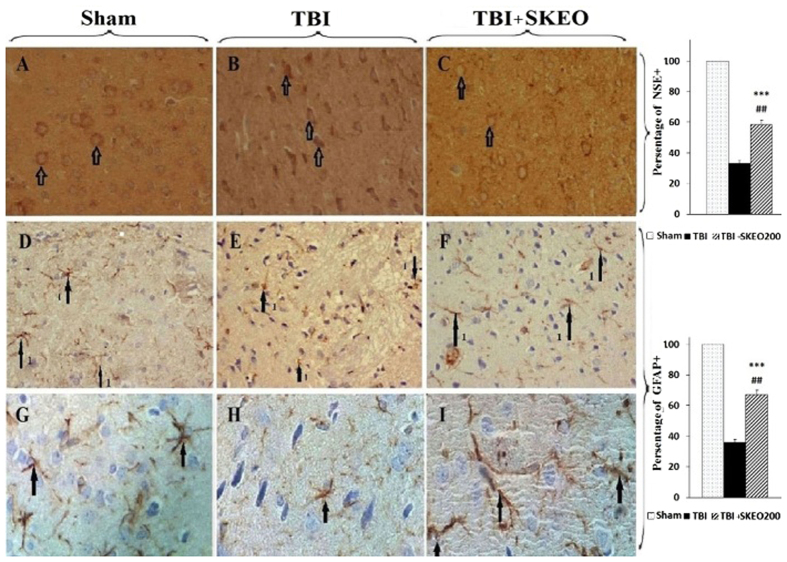
(**A**–**C**) IHC staining for NSE in the rat brain, (400X). (**A**) Sham; arrow indicate the normal neurons. (**B**) 24 hr after TBI; arrow indicate the degenerated neurons. (**C**) TBI+SKEO200; arrow indicate the viable neurons. (**D**–**I**) IHC staining for GFAP in the rat brain. In the sham group, arrows show normal astrocytes. 24 hours after TBI, arrows show degenerated astrocytes. In the TBI+SKEO200 group, arrows show normal astrocytes. (**D**–**F**: 400X, **G**–**I**: 1000X). The right panels illustrate the means ± SEM percentages of neuron-specific enolase (NSE)+ and glial fibrillar acidic protein (GFAP)+ cells from different groups. ***p < 0.001 compared to the sham group, ^##^p < 0.01 compared to the TBI group.

NSE-IHC staining revealed a significant increase in the population of non-damaged neurons in the TBI+SKEO200 group (58.485 ± 3.1%) compared with the TBI group (33.5 ± 2.1%) (p < 0.01, Fig. 7). Numbers of viable astrocytes also increased after treatment with SKEO200, and GFAP staining was positive in the TBI+SKEO200 group (67.04 ± 2.93%) as compared with the TBI group (36 ± 2.93%) (p < 0.01, Fig. 7).

## Discussion

In this study, we reported that SKEO was able to significantly reduce brain oedema, the permeability of the blood-brain barrier, and intracranial pressure, as well as improve neurological scores in rats. We also observed that SKEO resulted in reduced levels of pro-inflammatory cytokines, TNF-α (−36%), IL-1β (−38%), and IL-6 (−24%), along with a 44% increase in the level of anti-inflammatory cytokine IL-10.

The inflammatory reaction is initiated and regulated by an array of pro- and anti-inflammatory cytokines. Cytokines are small, short-lived proteins that are produced by blood leukocytes and glial cells. The expression profile of each cytokine following brain injury can provide information about the extent of tissue damage^7^.

Some studies have reported the anti-inflammatory effects of the genus *Satureja*. Amanloo *et al.* have shown reductions in inflammatory oedema induced by carrageenan in the hind paw of rats after the administration of SKJ extract; these reductions are comparable to the effects of indomethacin^16^. Hajhashemi *et al.* reported the suppression of pain and oedema induced by carrageenan after treatment with *Satureja hortensis*^32^. Carvacrol (the main compound of SKEO) has been shown to reduce brain oedema after intracerebral haemorrhage (ICH) in mice^21^. The above findings are consistent with our finding of reduced levels of inflammatory cytokines after the administration of SKEO.

The interference of macrophages with traumatic brain injury^33^ and the augmentation of nitric oxide (NO) production in murine macrophages by substance P have been reported^34^. Increases in blood pressure and intracranial pressure after the induction of TBI by NO have also been reported^35^, and the inhibition of NO production can improve neurological outcomes in TBI^36^. Interestingly, SKEO reduced NO production in a line of murine macrophages^37^. This is in agreement with the findings of Uslu *et al.*, who demonstrated a functional anti-inflammatory mechanism of *Satureja hortensis* by reducing NO production in rhinosinusitis^38^. Furthermore, Li *et al.* have observed that carvacrol significantly suppressed the induction of neuronal nitric oxide synthase (nNOS) after traumatic neural injury^39^. The prevention of TNF-α induced oedema^40^; the reduction by approximately half of the oedema induced by substance P in rat paws; the inhibition of leukocyte infiltration^41^; the inhibition of the expression of iNOS, IL-1β, and COX-2; and the increase in the level and expression of the anti-inflammatory cytokine IL-10^42^ are among the biological functions of carvacrol. Therefore, in this study it is reasonable to assume that the innate anti-inflammatory properties of SKEO200 led to reduced ICP at 4 and 24 hr after TBI.

The increase in neurological scores found in this study shows that SKEO200 may have neuroprotective effects.

Other studies have shown high concentrations of extracellular glutamate after TBIs. Glutamate is known to damage cells and cause the release of excitotoxic molecules and the production of free radicals^9^. Studies have reported the protective effects of *Satureja macrostema*, including the reduction of Glutamate Pyruvate Transaminase (SGPT)^43^ and an increase in anti-oxidative capacity after *Satureja khuzistanica* administration^44^. Improvements seen in inflammatory bowel disease after treatment with SKEO provide further evidence to emphasize the anti-oxidative effects of SKEO^17^. Kaidi *et al.* reported reductions in caspase-3 levels in the spines of diabetic rats after treatment with *Satureja khuzistanica*^15^. In 2012, Peters *et al.* inhibited TRP (transient receptor potential) channels with carvacrol. These channels play an important role in calcium transport and neuronal death^45^. Yu *et al.* indicated that carvacrol enhanced neurological recovery from brain ischaemia in mice^46^. In addition to these previous findings, reductions in apoptosis and caspase-3 levels have been reported as mechanisms by which carvacrol ameliorates neural brain injury^39^. Kaidi *et al.* reported that SKJ200 improves motor coordination in diabetic rats^15^. These results support the observed improvements in neurological outcomes mediated by SKEO treatment after TBI.

In the second part of this study, we observed decreases in blood cell infiltration following SKEO200 treatment in the brains of post-TBI animals (Figs 3 and 6), as well as increases in the viability and stability of astrocytes and neurons (Fig. 7). We may conclude that astrocytes, neurons and blood origin-infiltrated cells are the cytokines-producing sources.

Injury to the BBB results in oedema and abnormal water accumulation in the brain parenchyma. Water entering astrocytes from the extracellular space causes uncontrollable swelling of astrocytes within hours, as well as neuronal swelling. Hence, blood flow in small brain vessels is dramatically damaged. BBB injury causes blood cells such as neutrophils, lymphocytes, monocytes, and macrophages to enter the central nervous system^47^. These activated cells release intermediaries such as prostaglandins, free radicals, and inflammatory cytokines that induce chemokines, adhesion molecules, leading to recruitment of immune cells and activation of glial cells in the injured area^5^.

SKEO reduces the infiltration of blood cells and oedema by influencing the processes that occur following BBB breakdown (Figs 3 and 4). This conclusion is supported by our water content measurements and EB experiments (Fig. 1B,D).

As a mediator, TNF-α plays a significant role in the release of IL-6 and IL-1β by T-cells and other inflammatory cells^48^. TNF-α is also produced by microglia and astrocytes^49^. The release of IL-6 causes neuronal damage, blood-brain barrier damage, and acute neurological complications^50^.

Our data show that SKEO200 attenuates neuronal death as well as increases the stability of astrocytes (Fig. 7). This may be due to a reduction in TNF-α production. Esmaili Mahani *et al.* have shown that SKJ reduces TNF-α levels in rat spinal glial cells^51^.

The complex interactions between cytokines and cell types create a series of events that intersect with adjacent pathological cascades including oxidative stress, excitotoxicity, and reparative events comprising angiogenesis, scarring, and neurogenesis^7^.

Ultrastructural determination of the effect of expansive contusions on neurons depends on events such as perivascular haemorrhage^52^, astrocytic swelling^53^, the activation of macrophages, and apoptosis^54^. Damage to the BBB and astrocyte injury in TBI are correlated, and higher levels of IL-6 correspond with increased inflammatory response after TBI, as higher levels of astrocyte marker protein correlate with changes to the foot structures of astrocytes and glia^49^.

The stabilization of haemostasis is an important protective measure that reduces cell death in biological systems^55^. The inflammatory mediator bradykinin has been reported to activate calcium channels in glial cells and astrocytes. This activation sends signals to neighbouring neurons through the release of glutamate^56^. Carvacrol not only suppresses apoptosis but also prevents calcium influx induced by bradykinin^39^. Carvacrol has also been found to impede the expression of pro-inflammatory cytokines and cell apoptosis in neuroblastoma cells^57^.

Incorporating these findings, it can be inferred that SKEO decreases the magnitude of injury to neurons, astrocytes and the BBB through multistage effects. On the other hand, SKEO impedes the production of pro-inflammatory cytokines from these sources and results in dramatically decreased brain damage. Immunohistochemical results support these effects.

## Conclusion

This study showed that the SKEO prevents complications of TBI through reductions in brain oedema, prevention of damage to the BBB, reductions in ICP, and improvements in neurological scores. These effects may be mediated by reductions in the levels of inflammatory cytokines such as IL-1β, TNF-α, and IL-6 or by increases in the levels of anti-inflammatory cytokine IL-10, resulting in protection of the BBB, astrocytes, and neurons in this model of TBI. Investigation of the detailed mechanisms by which SKEO protects against traumatic brain injury requires further study.

## Additional Information

**How to cite this article**: Abbasloo, E. *et al.* The anti-inflammatory properties of *Satureja khuzistanica* Jamzad essential oil attenuate the effects of traumatic brain injuries in rats. *Sci. Rep.* **6**, 31866; doi: 10.1038/srep31866 (2016).

## Figures and Tables

**Table 1:** Composition of *Satureja khuzistanica* Jamzad essential oil.

Compound	RI^1^	Composition (%)	Identification^2^
Carvacrol	1282	94.16 ± 0.46	RI, MS, Col
*p*-Cymene	1017	0.96 ± 0.86	RI, MS, Col
*γ*-Terpenene	1053	0.51 ± 0.23	RI, MS, Col
(Z)- *β*-Oeimene	1036	0.42 ± 0.08	RI, MS
*α*-terpinole	1175	0.32 ± 0.45	RI, MS
Myreene	981	0.21 ± 0.19	RI, MS
*α*-Terpinene	1013	0.18 ± 0.12	RI, MS, Col
*α*-Thujene	925	0.14 ± 0.14	RI, MS
*α*- Pinene	933	0.12 ± 0.05	RI, MS, Col

RI^1^; Retention indices determined relative to n-alkanes (C_6_-C_24_) on a DB-5GC column. RI; Retention indices, MS; massspectra, Col; co-injection.
