# Erratum: The anti-inflammatory properties of *Satureja khuzistanica* Jamzad essential oil attenuate the effects of traumatic brain injuries in rats

**DOI:** 10.1038/srep33769

**Published:** 2016-09-21

**Authors:** Elham Abbasloo, Fatemeh Dehghan, Mohammad Khaksari, Hamid Najafipour, Reza Vahidi, Shahriar Dabiri, Gholamreza Sepehri, Gholamreza Asadikaram

Scientific Reports 6: Article number: 3186610.1038/srep31866; published online: 08 18 2016; updated: 09 21 2016

The original version of this Article contained a typographical error in the spelling of the author Gholamreza Asadikaram, which was incorrectly given as Golamreza Asadikaram. This has now been corrected in the PDF and HTML versions of the Article.

